# Inflammasome Activation Induces Pyroptosis in the Retina Exposed to Ocular Hypertension Injury

**DOI:** 10.3389/fnmol.2019.00036

**Published:** 2019-03-13

**Authors:** Alexey Pronin, Dien Pham, Weijun An, Galina Dvoriantchikova, Galina Reshetnikova, Jianzhong Qiao, Zhanna Kozhekbaeva, Ashlyn E. Reiser, Vladlen Z. Slepak, Valery I. Shestopalov

**Affiliations:** ^1^Department of Molecular and Cellular Pharmacology, University of Miami Miller School of Medicine, Miami, FL, United States; ^2^Bascom Palmer Eye Institute, Department of Ophthalmology, University of Miami Miller School of Medicine, Miami, FL, United States; ^3^Department of Medicine, The Division of Hematology and Oncology, University of Miami Miller School of Medicine, Miami, FL, United States; ^4^Geisinger Commonwealth School of Medicine, Scranton, PA, United States; ^5^Institute for Information Transmission Problems, Russian Academy of Sciences, Moscow, Russia; ^6^Department of Cell Biology, University of Miami Miller School of Medicine, Miami, FL, United States

**Keywords:** retina, inflammasome, mechanical stress, ischemia, pannexins, caspase-1 (ICE), pyroptosis

## Abstract

Mechanical stress and hypoxia during episodes of ocular hypertension (OHT) trigger glial activation and neuroinflammation in the retina. Glial activation and release of pro-inflammatory cytokines TNFα and IL-1β, complement, and other danger factors was shown to facilitate injury and loss of retinal ganglion cells (RGCs) that send visual information to the brain. However, cellular events linking neuroinflammation and neurotoxicity remain poorly characterized. Several pro-inflammatory and danger signaling pathways, including P2X7 receptors and Pannexin1 (Panx1) channels, are known to activate inflammasome caspases that proteolytically activate gasdermin D channel-formation to export IL-1 cytokines and/or induce pyroptosis. In this work, we used molecular and genetic approaches to map and characterize inflammasome complexes and detect pyroptosis in the OHT-injured retina. Acute activation of distinct inflammasome complexes containing NLRP1, NLRP3 and Aim2 sensor proteins was detected in RGCs, retinal astrocytes and Muller glia of the OHT-challenged retina. Inflammasome-mediated activation of caspases-1 and release of mature IL-1β were detected within 6 h and peaked at 12–24 h after OHT injury. These coincided with the induction of pyroptotic pore protein gasdermin D in neurons and glia in the ganglion cell layer (GCL) and inner nuclear layer (INL). The OHT-induced release of cytokines and RGC death were significantly decreased in the retinas of Casp1^−/−^Casp4(11)^del^, Panx1^−/−^ and in Wild-type (WT) mice treated with the Panx1 inhibitor probenecid. Our results showed a complex spatio-temporal pattern of innate immune responses in the retina. Furthermore, they indicate an active contribution of neuronal NLRP1/NLRP3 inflammasomes and the pro-pyroptotic gasdermin D pathway to pathophysiology of the OHT injury. These results support the feasibility of inflammasome modulation for neuroprotection in OHT-injured retinas.

## Introduction

Hypoxia and mechanical stretch during episodes of ocular hypertension (OHT) induce neuroinflammation in the retina and optic nerve. Neuroinflammation is marked by activation of microglia and astrocytes, which is linked to the release of pro-inflammatory cytokines (Yang et al., 2011; Krizaj et al., 2014; Lim et al., 2016; Lu et al., 2017), other danger factors and deposition of C1q complement on neuronal dendrites (Williams et al., 2016; Liddelow et al., 2017). Neuroinflammatory damage that includes dendritic pruning, axonal injury and neurotoxicity was shown to facilitate injury and loss of retinal ganglion cells (RGCs) in the OHT-injured retina (Soto and Howell, 2014). Recent studies, performed in the glia-neuron co-cultures (Orellana et al., 2012; Liddelow et al., 2017) indicated that cytokines released by glia play a major role in such damage. Consistently, blockade of different pro-inflammatory signaling resulted in RGC protection (Dvoriantchikova et al., 2009; Yang et al., 2011; Howell et al., 2014; Krizaj et al., 2014; Madeira et al., 2015; Silverman et al., 2016; Williams et al., 2016). Pro-inflammatory cytokines, including the IL-1 family, are produced by glial, microglial and neuronal cells in the retina in response to ischemic injury and in glaucoma (Yoneda et al., 2001; Ivanov et al., 2006, 2008; Namekata et al., 2009; Qi et al., 2014). Their production is controlled by inflammasome activation and significantly increased after exposure to OHT (Barakat et al., 2012; Chi et al., 2014). Genetic ablation and/or pharmacological inhibition of caspase-1 or inflammasome regulators, including Panx1, IL-1 or P2X receptors was shown to protect retinal neurons in several injury paradigms (Yoneda et al., 2001; Zhang and Chintala, 2004; Arai et al., 2006; Pelegrin et al., 2008; Seki et al., 2009; Dvoriantchikova et al., 2012; Puyang et al., 2016).

Sterile injury-induced inflammasome activation has been demonstrated in neural cells of the central nervous system (CNS; de Rivero Vaccari et al., 2008, 2009; Abulafia et al., 2009). Activation of both canonical and non-canonical inflammasomes was documented in the inner retina and optic nerve of rodent eyes challenged with transient elevation of intraocular pressure (IOP; Dvoriantchikova et al., 2012; Chi et al., 2014; Qi et al., 2014; Albalawi et al., 2017). However, temporal and cellular expression patterns of inflammasome pathways in the retina exposed to the OHT injury remain poorly characterized. Canonical inflammasomes, assembled by the NLRP1, NLRP3 and Aim2 sensor proteins, proteolytically activate caspases 1 and/or 11 (caspase-4 in humans) that are essential for processing and release of IL-1β. This is typically marked by oligomerization of the apoptosis-associated speck-like (ASC) adaptor protein into a megacomplex with NLRPs/Aim2 sensors and caspase-1 to form ASC “specks.” The most recent research showed that, in addition to IL-1 precursors, caspases 1 or 11 also cleave the N-terminal pore-forming part of gasdermin D protein (GSDMD-NT). The GSDMD-NT fragments oligomerize to form membrane megapores that release IL-1 but could become cytolytic and cause pyroptotic cell death (Aglietti et al., 2016; Chen et al., 2016; Liu et al., 2016; Sborgi et al., 2016). Although inflammasome-induced pyroptosis have been shown to contribute to pathogenesis of age-related macular degeneration (Tseng et al., 2013; Brandstetter et al., 2016; Viringipurampeer et al., 2016; Kerur et al., 2018), potential role of pyroptosis in OHT injury has not been investigated so far.

Pannexin1 (Panx1) has been implicated in the pathophysiological cascade triggered by the OHT injury (Thompson et al., 2006; Bargiotas et al., 2009), as well as in inflammasome activation in the CNS and the retina (Dvoriantchikova et al., 2006, 2012; Silverman et al., 2009; Adamson and Leitinger, 2014; Beckel et al., 2014). In the retina, Panx1 is abundant in RGCs, where its expression level correlates with RGC sensitivity to ischemic and mechanical damage (Dvoriantchikova et al., 2018). Panx1 interactions with various membrane receptors underlie its responsiveness to a variety of common neurotoxic and danger signals (Krizaj et al., 2014; Makarenkova and Shestopalov, 2014). While TNF, TLR4 and IL-1 receptor-signaling regulate transcriptional “priming” of inflammasome components (Signal 1), the signaling *via* Panx1-P2X7 signalosome regulates their assembly (Signal 2), which is critically required for activation (Zhang and Chintala, 2004; Silverman et al., 2009; Yang et al., 2011; Krizaj et al., 2014; de Rivero Vaccari et al., 2014). The Panx1-P2X7 signalosome was shown to play a central role in the increase of extracellular ATP and dysregulation of intracellular Ca^2+^ and K^+^, the key inflammasome-triggers in the CNS and retinal injuries such as the mechanical/ischemic insult during the OHT injury (Krizaj et al., 2014; Makarenkova and Shestopalov, 2014).

What cell types are known to activate inflammasome in the post-ischemic or mechanically injured retina? Currently, a growing number of reports indicate that retinal pigment epithelium (RPE; Anderson et al., 2013; Brandstetter et al., 2015; Gelfand et al., 2015), astrocytes (Albalawi et al., 2017), Muller cells (Devi et al., 2012; Mohamed et al., 2014; Natoli et al., 2017) and microglia (Abulafia et al., 2009; Ystgaard et al., 2015) can activate NLRP3 inflammasome. Mixed retinal glia cultures responded robustly to ATP stimulation after priming by *E. coli* lipopolysaccharide (LPS; Murphy et al., 2012). Muller cells were shown to produce IL-1β under hyperglycemic conditions (Busik et al., 2008; Devi et al., 2012), photo-oxidative injury (Natoli et al., 2017) and in amyloid beta toxicity models (Dinet et al., 2012). Both astrocytes and Muller cells were reported to cause neurotoxicity after stimulation with activated microglia in various disease models (Natoli et al., 2017; Yun et al., 2018). However, relative activation of inflammasome and production of IL-1 cytokines is reportedly more robust in retinal astrocytes and Muller cells, rather than in microglia (Li et al., 2012; Ystgaard et al., 2015), suggesting that macroglial cells are the major drivers of neuroinflammatory damage in the inner retina.

In this work, we sought to determine spatial and temporal patterns of inflammasome activation after OHT injury. We detected that activation of three canonical inflammasomes, NLRP1, NLRP3 and Aim2 in retinal glia, microglia and RGCs is co-regulated by Panx1. To explore which danger signals produce the most robust activation, we applied defined inflammasome inducers to mouse retinas *in vivo* using intravitreal injection. Our results picture a complex pattern of neuron-glia interactions that underlie innate immune responses in OHT injury and facilitate the injury-induced neurotoxicity.

## Materials and Methods

### Animals and Treatments

All experiments and postsurgical care were performed in compliance with the National Institutes of Health (NIH) Guide for the Care and Use of Laboratory Animals and according to the University of Miami IACUC approved protocol #18-031. Wild-type (WT) animals used in our experiments were 2–4-month-old male mice of the C57BL/6 background. Mice were bred and maintained in the University of Miami animal facility and housed under standard conditions of temperature and humidity with a 12-h light/dark cycle and free access to food and water. The Panx1^−/−^ mouse line was generated as described previously (Dvoriantchikova et al., 2012) and extensively characterized (Tordoff et al., 2015) thereafter. An alternative strain of Panx1^−/−^ animals with full zygotic ablation of protein in mice with a B6 genetic background [Panx1^−/−^/B6, developed by V.M. Dixit (Qu et al., 2011)], was obtained from Genetech Inc. (Oceanside, CA, USA) and backcrossed with C57BL/6 for minimum of 7 generations. Casp1^−/−^ Casp4(11)^del^ mice were obtained from the depository at Jackson Laboratories (strain B6N.129S2-Casp1tm1Flv/J). Mouse strains with full ablation of Casp11 on the C57Bl/6 background [referred to as Casp11^−/−^, Jax strain B6.129S4(D2)-Casp4tm1Yuan/J] possessing WT Panx1, were purchased from Jackson Laboratory (Bar Harbor, ME, USA). The bioindicator mice expressing ASC fusion protein with a C-terminal Citrin (fluorescent GFP isoform), which allowed for the visualization of filamentous ASC “specks,” were provided by D. Golenbock (University of Masachussetts, MA, USA).

Intraocular injections of drug inhibitors and inflammasome-inducer cocktails were performed in a total volume of ≤2.0 μl using a G35 needle to minimize potential injury site and recovery time. The contralateral control eye received carrier (PBS or DMSO/PBS mix) without IOP elevation; naïve eyes were used as controls for systemic responses induced by unilateral injury. Probenecid was injected intraperitoneally 1 h prior to IOP elevation at 2.0 mM in sterile PBS as described (Mawhinney et al., 2011). Samples for IL-1β release and GSDMD expression studies were pooled from a group of 3–5 animals aged 2–4 months with a minimum of three biological repeats per treatment. The following inflammasome agonists and working concentrations were used for intraocular injections: from InVivogen, LPS-EB ultrapure (100 μg/ml, ID#tlrl-3pelps), ultrapure *S. typhimurium* flagellin (FLA-ST; 25 ng/ml, ID# tlrl-epstfla), nigericin (10 μg/ml, ID#tlrl-nig) and poly dA:dT synthetic DNA(15 μg/ml, ID# tlrl-patn); from Tocris, bzATP (100 μM, ID# 3312). For intracellular delivery, poly dA:dT synthetic DNA was mixed with Lipofectamine 2000 Transfection Reagent Thermo Fisher Scientific (ID# 11668027).

### Cytokine Activity Assays

ELISA kits (IDs# ab197742) were used to measure IL-1β release in mouse vitreous. Following perfusion with PBS, mouse eyes were placed on ice and immediately dissected to obtain vitreous fluid. Three consecutive 25 μl flushes of the vitreous cavity with sterile PBS/protease inhibitor cocktail were combined with vitreous body fluid, spun for 5 min in a refrigerated centrifuge and stored at −80°C. The aliquots were processed for ELISA in parallel with standards and controls following the manufacturer’s instructions. Colorimetric assay was done using a FLUOstar Omega plate reader (BMG Labtech) and analyzed using MARS data analysis software (BMG Labtech). Values from the wells containing blank samples were subtracted as the background. To validate the significance of measurements at the lowest reading, the limit of detection (LOD) and limit of quantification (LOQ) ratios were calculated from empirical data obtained in the “zero” wells of each plate, as described (Shrivastava and Gupta, 2011). The minimum of three (*N* ≥ 3) biological repeats were used for each data point. Significance was calculated using one-way analyses of variance (ANOVA) followed by Tukey’s test for multiple comparisons.

### Acute Ocular Hypertension (OHT) Injury Models

Transient retinal OHT (ischemia-reperfusion) was induced as previously described (Dvoriantchikova et al., 2009; Barakat et al., 2012). Prior to applying the isoflurane gas anesthesia, pupils were dilated with 1% tropicamide-2.5% phenylephrine hydrochloride (NutraMax Products, Inc., Gloucester, MA, USA); one drop of 0.5% proparacaine HCl (Bausch and Lomb Pharmaceuticals, USA) per eye was applied for corneal analgesia. Retinal ischemia was induced by increasing IOP above systolic blood pressure (to 120 mm Hg) for 45 min. IOP was elevated by direct cannulation of the anterior chamber of the eye with a 33-gauge needle attached to a normal (0.9% NaCl) saline-filled reservoir raised above the animal. The contralateral eye was cannulated and maintained at normal IOP to serve as a normotensive control. Complete retinal ischemia, evidenced by a whitening of the anterior segment of the eye and blanching of the retinal arteries, was verified by microscopic examination. After needle removal, erythromycin ophthalmic ointment (Fougera and Atlanta, Inc., Melville, NY, USA) was applied to the conjunctival sac. Mice were sacrificed at experimental time points by CO_2_ inhalation under anesthesia.

### *In situ* RNA Hybridization

*In situ* RNA hybridization was performed using RNAscope technology (Advanced Cell Diagnostics, Hayward, CA, USA) following the manufacturer’s protocol, as described (Pronin et al., 2014). Briefly, at 12 h postinjury, experimental and control eyes were dissected, formalin fixed and embedded in optimal cutting temperature (OCT) medium. Whole-fixed mouse eyes were cut into 10 μm sections and mounted on SuperFrost Plus glass slides. After removing the OCT medium with PBS, slides were treated for 15 min with boiling pretreatment 2 solution, followed by pretreatment 3 (protease) for 30 min at 37°C. To reduce chromosomal DNA background, we introduced a DNase treatment step: slides were washed 5× with water and treated with DNase I (50 U/ml in 1× DNase I buffer, Ambion) for 40 min at 37°C. To demonstrate that the signal comes from hybridization of probes with mRNA, some slides were treated with a mixture of DNase I and RNase A (5 mg/ml). Following 5× wash with water, slides were hybridized with RNAscope probes for 2 h at 40°C. Following *in situ* RNA hybridization steps, regular immunohistochemistry was performed to colocalize transcript signals with retinal cell markers: RBPMS for RGCs, GFAP for astrocytes, glutamine synthetase for Muller glia and Iba1 for microglial cells. The fluorescent signals were visualized and captured using a Leica SP5 confocal microscope.

### Real-Time PCR Analysis

Gene expression was assessed by real-time PCR (RT-PCR), using gene-specific primer pairs for mouse gasdermin D (Gsdmd) gene Gsdmd-F: TCATGTGTCAACCTGTCAATCAAGGACAT and Gsdmd-R: CATCGACGACATCAGAGACTTTGAAGGA. For the quantitative PCR this pair of primers was validated to span an intron and to amplify only one product. Total RNA was extracted using the Absolutely RNA Nanoprep kit (Agilent Technologies, Wilmington, DE, USA) and reverse transcribed with the Reverse Transcription System (Promega, Fitchburg, WI, USA). RT-PCR was performed in the Rotor-Gene 6000 Cycler (Corbett Research, Mortlake, Australia) using the SYBR GREEN PCR MasterMix (Qiagen, Valencia, CA, USA). Relative transcript abundances were calculated by comparison with a standard curve following normalization to the mouse Gapdh gene. The minimum of three biological repeats were used for each data point.

### Immunohistochemistry

Eyes were enucleated, fixed in 4% paraformaldehyde for 30 min, and cryoprotected with 30% sucrose. Whole eyes were frozen and then sectioned to a thickness of 10 μm on a Leica cryotome (Germany). After washing, permeabilization and blocking, sections were incubated with a primary antibody for 4–16 h. Retinal flat-mounts were incubated with primary antibodies for 3–5 days at 4°C to ensure even staining across retinal layers. AlexaFluor dye-labeled and secondary antibodies (Thermo Fisher Scientific, Waltham, MA, USA) were applied for imaging. The following commercially available antibodies were used: neuronal Brn3a (Santa Cruz), class III β-tubulin (TUJ-1, Covance ID # MMS-435P), RBPMS (GeneTex ID# GTX118619), NeuN (Invitrogen ID#702022); glutamine synthetase (GeneTex ID# GTX109121); IL-1b (Cell Signaling ID#8689); Iba1 (Wako/FUJI ID# 019-19741), GFAP (Dako, cat#z0334), CD11b (Biolegend ID#101218), Casp1 (Novus Biologicals ID#NB100-56565R), Casp11 (Novus Biologicals ID# NB120-10454), ASC (Adipogen ID#AG25b-0006), NLRP1 (Novus Biologicals ID #NB100-56148), NLRP3 (Adipogen ID# AG20b-0014-C100), Aim2 (Boster Biological Technology ID#PB9683), GSDMD (Santa Cruz ID#sc-393656). Species-specific secondary fluorescence AlexaFluor dye-conjugated antibodies for confocal microscopy were purchased from Invitrogen/Molecular Probes, USA.

### Western Blot Assay

Mouse retinas were isolated from enucleated eyes and dissolved in 200 μl of 1× T-PER tissue protein reagent (Thermofisher ID#78510) buffer. Lysates were homogenized and briefly sonicated (to destroy chromosomal DNA) and resolved on SDS-polyacrylamide gels, followed by immunoblotting using antibodies against proteins of interest. The secondary antibodies labeled with infrared IRDye 800CW or 680RD were from LI-COR, Inc. The immune complexes were visualized using an Odyssey (LI-COR) infrared fluorescence detection system, and for quantitative analysis, the signal in the band of interest was normalized to the signal for actin in the same lane.

### Analysis of RGC Loss

RGC loss in the inner retina was assessed by direct cell counting using immunolabeling with a neuron-specific NeuN staining in the ganglion cell layer (GCL) layer. Whole retinas were flat-mounted and coverslipped, and specific fluorescence in the inner retina was imaged. To avoid topological irregularities, stacks of five serial images collected at a depth of 0–30 μm were collapsed to generate the “maximum projections” (standard feature of the Leica LAS AF software), where all imaged cells appear in sharp focus. These images were used for RGC counts with Image J (NIH, USA) software, after image thresholding and manual exclusion of artifacts. Individual retinas were sampled at 20 random fields in three regions/four retinal quadrants at the same eccentricities (5 at 0.5 mm, 10 at 1.0 mm and 5 at 1.5 mm from the optic disk) using a 20× objective lens as we reported earlier (Dvoriantchikova et al., 2012). RGC loss was calculated as the percentages of βIII-tubulin-positive cells in experimental eyes relative to sham-operated contralateral control eyes that were cannulated but maintained at normal IOP. The data from a minimum of five animals were averaged for each group/genotype.

### Caspase-1 Activity Detection

The assays were performed using Caspase1 FLICA 660-YVAD-FMK detection kit (Immunochemistry Technologies Inc., ID#9122). Labeled caspase-1 substrate was added to the culture medium or injected intravitreally *in vivo* 1 h prior to cell fixing or animal termination. Cells and tissues were processed for imaging according to the manufacturer protocols. Caspase-1 positive cells were imaged (590/660 nm excitation/emission) by confocal microscopy.

#### Statistical Analysis

Data are presented as the mean ± standard deviation (SD) for gene expression and ELISA data or standard error (SEM) for RGC survival data. GraphPad Prism software (version 6.07; GraphPad Software, La Jolla, CA, USA) was used for statistical analysis. The minimum of three biological repeats per treatment were used for *in vivo* IL-1β release assessment and for gene expression analysis by quantitative RT-PCR. Groups of data were compared using ANOVA or two-tailed unpaired Student’s *t*-tests, with values of *P* < 0.05 considered statistically significant. The cell density data were analyzed with one-way ANOVA followed by Tukey’s test for multiple comparisons. For single comparisons, Student’s *t*-test was applied. *P* values < 0.05 were considered statistically significant.

## Results

**Figure 1:**
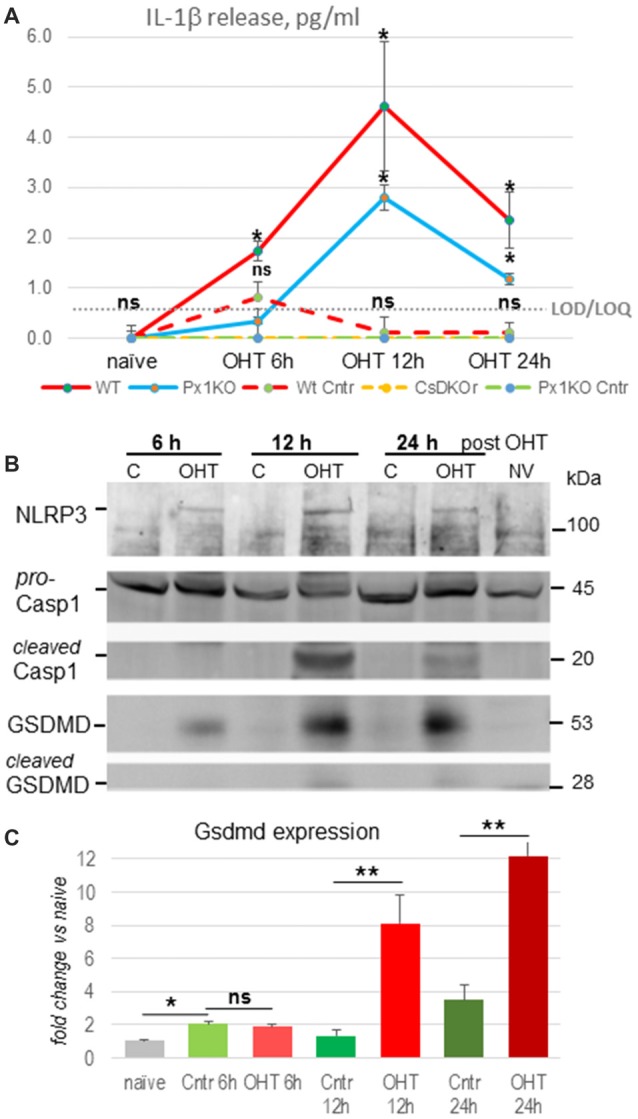
Inflammasome activation after ocular hypertension (OHT) injury. **(A)** IL-1β cytokine release from OHT-challenged retina measured in naïve eyes, normotensive control (dotted lines) and experimental eyes (solid lines) at 6, 12 h and 24 h after injury in C57Bl6/J [Wild-type (WT), red], Panx1^−/−^(Px1KO, blue) and Casp1^−/−^Casp4(11)^del^ (CaspDKO, dotted yellow) mice. Significant IL-1β release from control normotensive control eyes was only detected in WT animals (WT Cntr, dashed) at 6 h post-OHT; the zero level release from Px1KO and CaspDKO is shown as green dotted line. Gray dotted line represents limit of detection (LOD)/limit of quantification (LOQ) value of 0.58 ± 0.036 for these series of experiments. Statistics: mean ± standard deviation (SD); *P < 0.05; ns, no significance; *N* = 6. **(B)** Western blot analysis of NLRP3, Casp1 and gasdermin D proteins in control and experimental WT retinas at 6, 12 and 24 h post-OHT retinas. Untrimmed gel images: Supplementary Figure S4. **(C)** Changes in GSDMD gene expression in post-OHT retinas at 6, 12 and 24 h postinjury was assessed by qRT-PCR relative to naïve and normotensive control (cntr) retinas (*N* = 3). Results are presented as fold change in Gsdmd transcript abundance, normalization to Gapdh. Statistics: mean ± SD, **P < 0.01, *T*-Test; ns, non-significant changes.

We induced unilateral acute OHT in eyes of WT, Panx1^−/−^ and Casp1^−/−^Casp4(11)^del^ mice lacking both caspase-1 and caspase-11 inflammasomes and analyzed vitreo-retinal extracts from experimental and contralateral normotensive controls using ELISA. We detected release of IL-1β cytokines in WT eyes starting at 1.73 ± 0.19 pg/ml 6 h postinjury, peaking at 4.62 ± 1.28 pg/ml in 12 h and declining to 2.36 ± 0.56 pg/ml at 24 h after OHT injury (*P* < 0.05 at all time points; Figure 1A, red line). Normotensive WT control eyes showed statistically significant IL-1β release of 0.69 ± 0.11 pg/ml only at 6 h postinjury, which is consistent with the reported bilateral effect of unilateral ischemic injury (Sapienza et al., 2016). Relative to the WT eyes, the OHT-induced IL-1β release in Panx1^−/−^ mice was approximately 2-fold lower at all time points, reached the maximum of 2.8 ± 0.25 pg/ml at 12 h and 1.12 ± 0.11 pg/ml at 24 h postinjury (Figure 1A, blue line). No IL-1β release was detected in normotensive control eye of Panx1^−/−^ (green dotted line) or Casp1^−/−^Casp4(11)^del^ mice (CaspDKO, Figure 1A, dotted yellow line). Consistent with these findings, Western blot analysis of retinal extracts from the OHT-injured eyes showed increased levels of the principle IL-1 convertase caspase-1 and the major pore-forming protein GSDMD as well as that NLRP3 sensor protein at 12 and 24 h postinjury (Figure 1B). Both Casp1 and GSDMD showed cleaved mature isoforms, the evidence of their proteolytic activation, at the same time points. To further validate the OHT-induced acute activation of GSDMD, we used RT-PCR to confirm gene activation. Our analysis detected robust increase of GSDMD expression, averaging 6.5- and 3.4-fold increase relative to normotensive control eyes (8.1- and 12.2-fold relative to naïve eyes) at 12 h and 24 h postinjury, respectively (Figure 1C). Noteworthy, the control eyes showed a significant 2.1- and 3.6-fold increase in GSDMD expression relative to naïve eyes at 6 h and 24 h postinjury.

**Figure 2:**
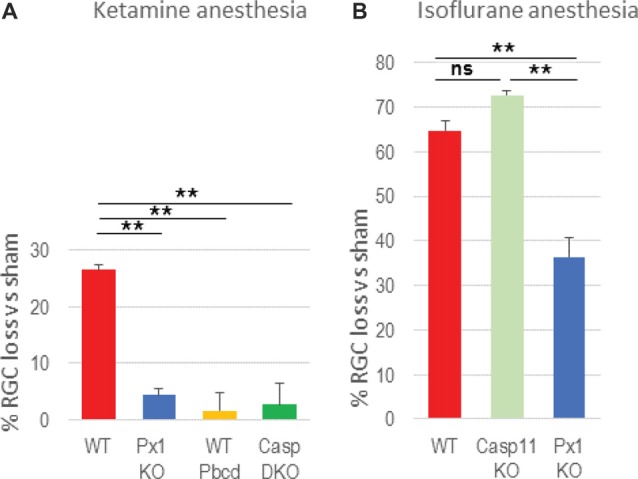
Blockade of inflammasome pathways suppresses retinal ganglion cell (RGC) loss in OHT. **(A)** RGC loss [NeuN^+^ cells in the ganglion cell layer (GCL)] was assessed as % change between experimental and normotensive control eyes of same animals after OHT injury. The loss in retinas with genetic ablation of pannexin-1 (Px1KO; 4.5 ± 5.1%, *N* = 11), of both caspases −1 and −11 (CaspDKO, 3.4 ± 3.4%, *N* = 5) was compared to that in WT (C57Bl6J, 26.4 ± 4.9%, *N* = 10) reference retinas and in WT mice with 2.0 mM probenecid treatment (WT Pbcd, 4.4 ± 6.0%, *N* = 5). **(B)** RGC loss after OHT injury performed under isoflurane gas anesthesia was assessed in the WT (64.7 ± 5.2%) retinas, Panx1^−/−^/Casp11^+/+^ retinas (41.1 ± 8.9%) and in the retinas with Caspase-11 ablation (72.7 ± 2.9%). Statistics: mean ± standard error (SEM), **P < 0.01; ns, no significance;significance: one-way analyses of variance (ANOVA) and Tukey test for multiple comparisons.

Next, we asked the question whether inflammasome activation contributes to neuronal loss in OHT injury. Using a gene knockout approach, we showed that ablation of the Casp1 gene protected RGCs (3.4 ± 3.4% vs 26.4 ± 4.9% loss in WT OHT retinas, Figure 2A). A similar effect was achieved by genetic ablation of the Panx1 gene (4.5 ± 5.1% loss), the upstream regulator of the inflammasome assembly. Importantly, a similar level of protection was achieved with pharmacological blockade of the Panx1 channel by the specific blocker probenecid (Pbcd). Noteworthy, Casp1^−/−^ mouse line from the Jackson Labs repository has the Casp11-inactivating Casp4(11)^del^ passenger mutation, known to block pyroptosis induced by intracellular LPS (Yang et al., 2011; Aglietti et al., 2016). To test whether caspase-11 is also involved in OHT-induced neurotoxicity, we measured RGC survival in injured retinas of the Casp11^−/−^ knockout strains, as well as in the Panx1^−/−^Casp1^+^ animals (obtained from Genentech, back-crossed to C57Bl6 background for seven generations) that have functional Casp4(11) gene. In these experiments, we applied isoflurane gas instead of ketamine/xylasine anesthesia to take advantage of an increased (from 26% to about 65%) post-IR injury RGC loss rate, which would allow us detecting even minor differences in protection. Casp11 KO mice showed a 72.7 ± 2.9% loss of NeuN-positive cells that was not statistically different from the loss in the WT (64.7 ± 5.2%) retina (Figure 2). The fact that similar levels of RGC protection were observed in both Panx1^−/−^ and Casp1^−/−^Casp4(11)^del^ animals (4.5 ± 5.1% and 3.4 ± 3.4%, respectively), while the Casp11^−/−^ animals lacked (72.7 ± 2.9% vs 64.7 ± 5.2% loss) protection, helped us to rule out active Casp11 involvement in post-OHT degeneration (Figure 2B).

**Figure 3:**
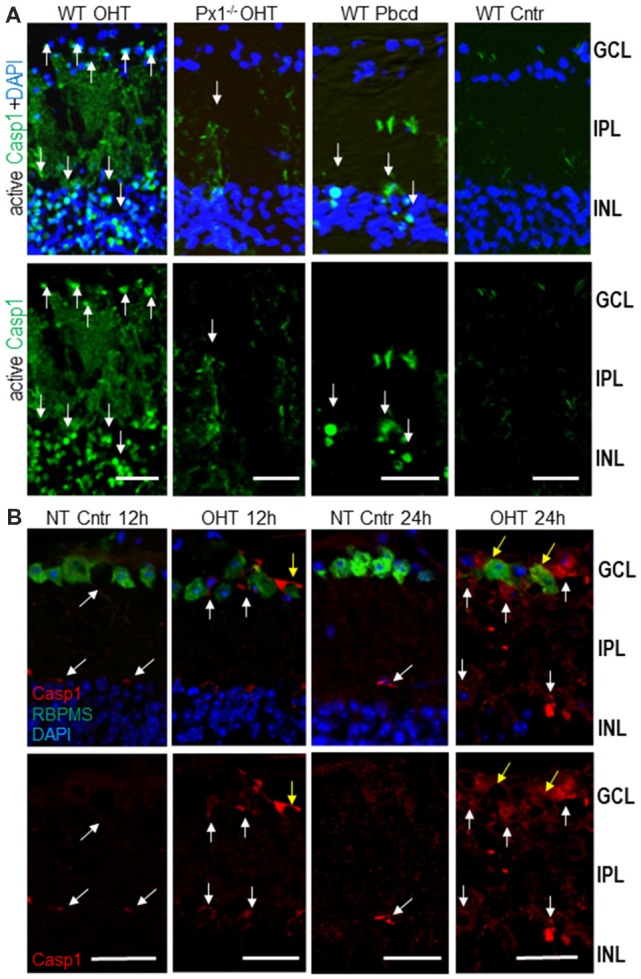
Activity of Casp1 in OHT-injured and normotensive control eyes.** (A)** Casp1 was detected by intraocular injection FLICA660-labeled substrate (green) *in vivo* 24 h after injury. Bright labeling (arrows) is evident in cells in the GCL and inner nuclear layer (INL) layers of the OHT-challenged retinas, a diffuse labeling of cell processes located in the IPL. Casp1 activity is diminished in Panx1^−/−^ (Px1^−/−^ OHT) retinas and WT retinas treated with probenecid (WT/Pbcd) at 12 h postinjury. **(B)** The analysis of the Casp1 immunolabeling (red, white arrows) in normotensive (NT control) and injured (OHT) retinas at 12 h and 24 h postinjury. Yellow arrows denote Casp1 colocalization with RGCs (RBPMS ^+^, green) as well as with other cells at 24 h post-OHT. Bar, 25 μm.

To map the activity of Casp1 interleukin convertase in retinal cells, we performed *in vivo* intravitreal injections of fluorescent Casp1 substrate FLICA 660-YVAD-FMK into both experimental (OHT) and normotensive control mouse eyes. The majority of Casp1-active cells localized within the inner nuclear layer (INL) and GCL layers of the inner retina (Figure 3A). As expected, Panx1 knockout or the treatment with the Panx1 blocker probenecid resulted in a dramatic suppression of Casp1 activity, particularly in the GCL, at 12 h post-OHT. Immunohistochemistry showed very low levels of Casp1 colocalizing to RGCs and their processes and retinal microvasculature in normotensive control eyes (NT Cntr, Figure 3B). In agreement with the activity mapping data, these levels were significantly increased in experimental OHT eyes of WT at 12 h and peaked at 24 h postinjury. Colocalization analysis showed the most intense specific staining for Casp1 protein in the GCL, colocalizing with RGCs (RBPMS^+^) and RBPMS^−^ neurons at 12 h and with astrocytes in the neurofiber layer at 24 h (Figure 3B).

**Figure 4:**
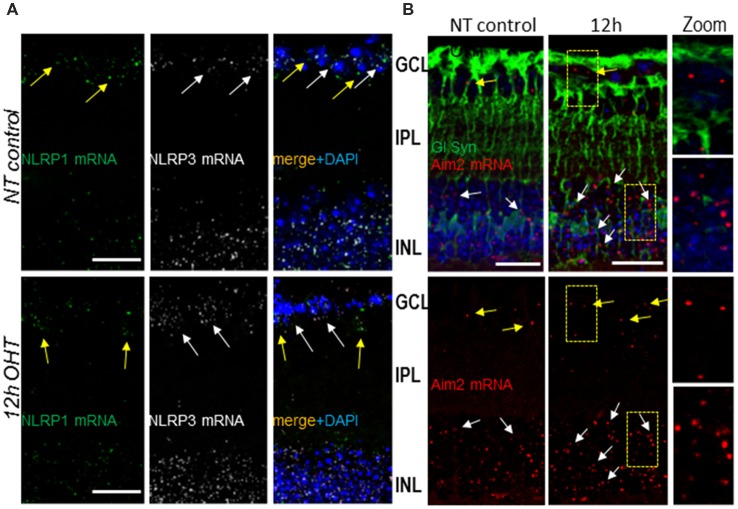
Gene expression and cellular distribution of NLRP1, NLRP3 and Aim2 sensors in the retina.** (A)** RNAscope analysis of NLRP1 (green dots, yellow arrows) and NLRP3 (white dots, white arrows) transcript abundance in normotensive control (NT control) and experimental (OHT) retinas at 12 h postinjury. **(B)** Aim2 transcript (red dots) abundance in normotensive control and experimental retinas. Co-staining for the Muller glia marker glutamine synthetase (GlSyn, green) is added to indicate cellular expression of Aim2 in the GCL layer (yellow arrows) and INL (white arrows) in control and 12 h post-OHT retinas. Bar, 25 μm on all panels.

Next, we investigated inflammasome sensor proteins potentially involved in proteolytic activation of Casp1 and release of IL-1β in OHT injured retinas. For this purpose, we examined expression of canonical NLRP1, NLRP3 and Aim2 using RNAscope mRNA *in situ* hybridization and immunostaining. RNAscope data analysis showed differential levels of expression of NLRP3 and Aim2 genes in the INL and GCL of OHT-challenged retinas relative to normotensive retinas at 12 h post-injury (Figure 4). In normotensive eyes, the expression of NLRP1 and NLRP3 genes (individual transcripts visualized as green and white dots, respectively) was observed in both GCL and INL layers. The NLRP3 and Aim2 gene transcripts were relatively abundant in the INL vs. GCL layers, while NLRP1 transcript showed similar abundance across the two layers (Figure 4). In the OHT-injured retinas, the NLRP3 and Aim2 transcripts showed an increase in abundance at 12 h after acute OHT injury compared to normotensive sham-treated control eyes. NLRP3 became more abundant in both GCL and INL (Figure 4A), Aim2 transcript had increased abundance only in the INL after OHT injury (Figure 4B), and NLRP1 remained unchanged.

To determine the cell types expressing distinct inflammasomes, we used multiplexing of the RNAscope labeling of transcripts with immunostaining for common glial cell markers: GFAP for astrocytes and glutamine synthetase (GlSyn) for Muller cells. In control normotensive retinas, the transcripts for NLRP1 colocalized predominantly with cells in the GCL, where astrocytes, microglia and two types of neurons, RGCs and displaced amacrine cells, are located. In both normotensive and experimental eyes 12 h after the OHT injury, the NLRP1 transcripts showed colocalization with cells in the INL and GCL, the retinal layer populated with astrocytes and RGCs (Supplementary Figure S1). Further analysis using co-immunostaining for GFAP marker protein showed no colocalization of NLRP1 transcripts with astrocytes (GFAP^+^ cells in the NFL later), in either normotensive control of OHT-challenged eyes (Supplementary Figure S1). The transcripts for gene encoding Aim2 sensor protein co-localized predominantly with GlSyn-positive Muller cells in the INL (Figure 4B).

**Figure 5:**
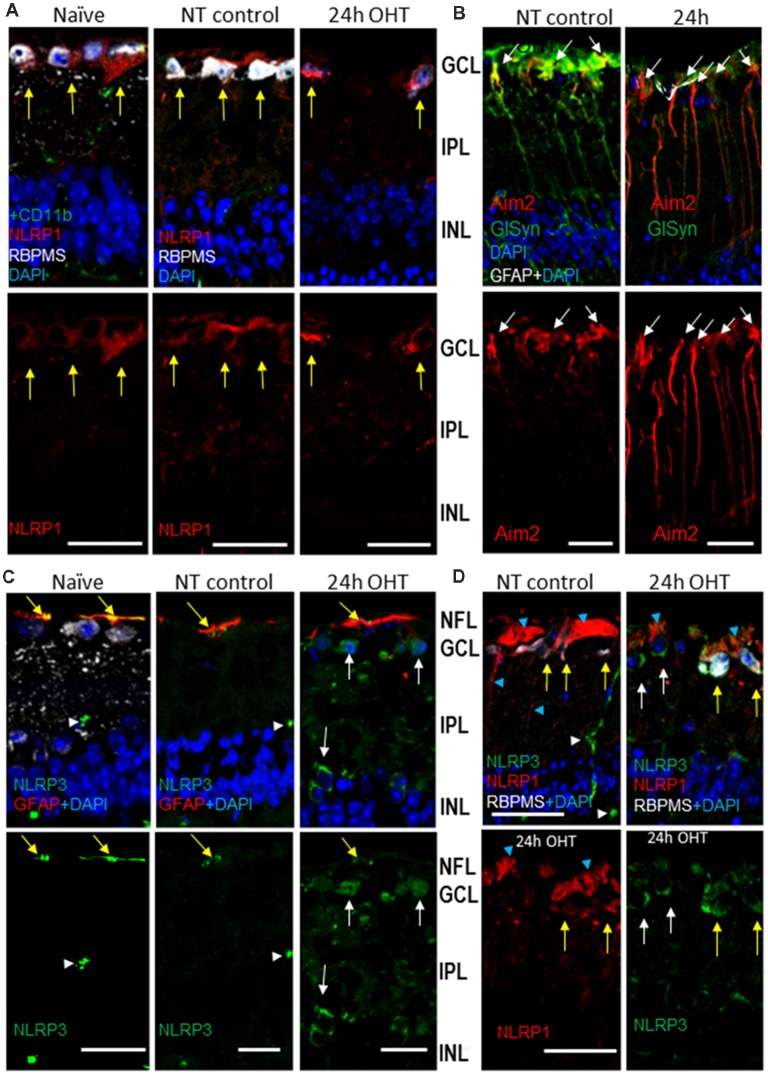
OHT-induced upregulation of Aim2 inflammasome in the retina.** (A)** NLRP1 antibody labeling (red) in the inner retina; yellow arrows indicate colocalization with RGC neurons (RBPSM^+^) in the GCL. **(B)** Immunohistochemistry for Aim2 protein (red) and Muller glia marker (GlSyn, green). Arrows show colocalization with Muller glia; no colocalization is detected with astrocytes (GFAP, white) in both control and 24 h post-OHT retinas. **(C)** NLRP3 labeling (green) in the inner retina of naïve and control eyes localized only to blood capillaries (white arrowhead) and astrocytes (yellow arrows), but not to RGCs (RBPMS cells). At 24 h post-injury the labeling in large cells in the GCL and INL (white arrows) are observed. **(D)** The NLRP1and NLRP3 proteins localized to distinct cell types in the GCL of naïve and NT control retinas: NLRP3 (green) is only expressed in blood vessels (white arrowheads). Twenty four hours after OHT, NLRP3 colocalizes with NLRP1 in RGCs (yellow arrows) and to RBPMS- cells (white arrows). Blue arrowheads denote labeling in RGC axons and dendrites. Bar, 25 μm on all panels.

Immunohistochemistry analysis showed labeling specific to the NLRP1 protein in the inner retina of normotensive eyes, where it predominantly colocalized with the RGC marker RBPMS (Figure 5A). The OHT challenge did not cause an increase in the NLRP1 labeling in these neurons and their axons in the NFL at 24 h post-insult (Figures 5A,D). NLRP1 labeling was also detected in axonal bundles in the NFL and in the RGC dendrites (Figures 5A,D). However, in contrast to NLRP1, the immunolabeling for NLRP3 protein has increased by 24 h post-OHT, colocalizing mostly with RGCs and RBPMS-negative neurons in the GCL and astrocytes in the NFL (Figures 5C,D). In both naïve an normotensive control retinas, NLRP3 labeling co-localized with GFAP^+^ astrocytes and, to a less degree, with CD11b^+^ microglial cells (Supplementary Figure S2A). No overlap was detected between NLRP3 and Aim2 labeling. Notably, in the outer retina, the highest NLRP3 labeling localized to the RPE (Supplementary Figure S2B). Immunohistochemistry showed strong colocalization of Aim2-specific labeling with the Muller glia marker GlSyn (Figure 5B). Combined, these data allow us to conclude that three canonical inflammasomes become activated early after OHT injury: NLRP1 and NLRP3 inflammasomes in the GCL, the NLRP3 inflammasome in astrocytes and the Aim2 inflammasome in Muller glia. Immunohistochemical evidence indicates expression of NLRP1 in RGCs of normotensive and naïve control retinas and expression of both NLRP1 and NLRP3 in these neurons after OHT challenge.

**Figure 6:**
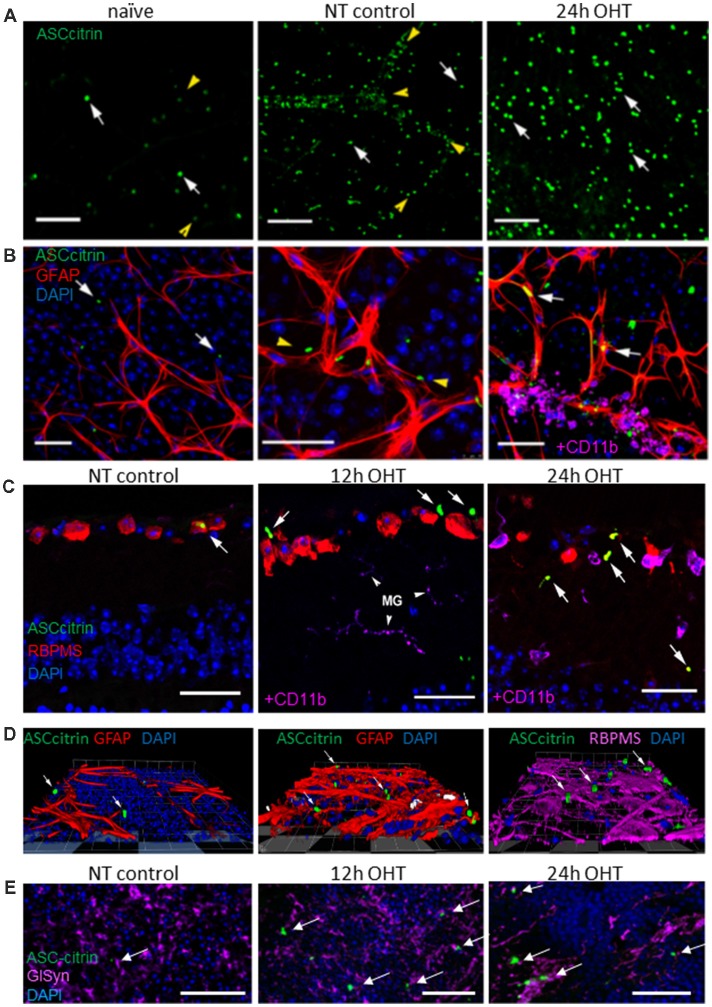
OHT injury caused an increase in apoptosis-associated speck-like (ASC) expression and ASC speck formation in the mouse retina.** (A)** Scarce ASC-citrin specks (green, white arrows) are detected in naïve retinas; the smallest specks were lined up along microvasculature (yellow arrowheads). In post OHT retinas, larger size specks were observed; normotensive control eyes had an increased speck density, particularly along blood vessels (arrowheads). **(B)** Immunostaining for astroglial cell marker (GFAP, red) revealed ASC speck (arrows) associated with astrocytes in OHT retinas, but not in naïve retinas. In control normotensive eyes ASC specks were localized in vasculature (arrowheads) ensheathed by astrocytes. At 24 h post-OHT, large specks are observed in astrocytes. Smaller specks colocalized with perivascular macrophages (CD11b, magenta). **(C)** Immunostaining in retinal slices shows ASC speck colocalization with RGCs (RBPMS, red) and microglia (CD11b, magenta) in OHT injured and control retinas. **(D)** Snapshots of 3D reconstructions showing ASC specks (arrows) colocalizing with astroglia (GFAP, red) and RGCs (RBPMS, magenta) in the GCL. **(E)** The analysis of retinal wholemounts showed ASC-citrin speck (arrows) at 45 μm depth show co-localizes with Muller glia marker GlSyn (magenta) in the INL at 24 h post-OT injury. Bar, 50 μm on all panels.

Next, we studied formation of the mature inflammasome complexes, referred to as ASC specks, which we visualized using a transgenic bioindicator model expressing fluorescent ASC-citrin fusion protein (Tzeng et al., 2016). ASC-citrin was shown to incorporate into oligomerizing inflammasome complexes to brightly label the ASC specks *in vivo*, thus providing a surrogate inflammasome activation marker in mouse tissues (Scheiblich et al., 2017; Venegas et al., 2017). Scarce small size ASC specks of incompletely assembled complexes colocalized primarily with glia-vascular interfaces in naïve retinas, a striking contrast to the large, mature ASC specks of active fully assembled complexes that were abundant in the GCL and INL of the OHT-challenged retinas (Figures 6A,B). Relative to naïve tissues, the normotensive control retinas had an increased speck abundance, with the most specks observed along blood vessels (Figure 6A), suggesting that the pro-inflammatory effect of the contralateral eye injury (Sapienza et al., 2016) is spread *via* systemic circulation. Immunostaining for cell markers in retinal sections showed occasional colocalization with astrocytes and RGCs in control normotensive eyes (Figures 6B,C), where specks aligned well with blood vessels ensheathed with astrocytes. In the post-OHT retina, a proportion of specks colocalized with astrocytes and CD11+ microglial/macrophage cells in NFL as well as with RBPMS-positive RGCs in the GCL (Figures 6B–D). The analysis of the INL showed ASC speck colocalization with GlSyn-positive Muller cells in retinal wholemounts, whose nuclei reside at approximately 40–45 μm deep below NFL layer (Figure 6E). The analysis of immunostaining for the ASC protein showed a robust induction at 24 h post-OHT but the pattern was different from ASC-citrin labeling, most likely because antibodies selectively recognized monomeric ASC. The labeling was observed in both RGCs, RBPMS-negative neurons in the GCL and in astrocytes in the NFL (Supplementary Figure S3).

**Figure 7:**
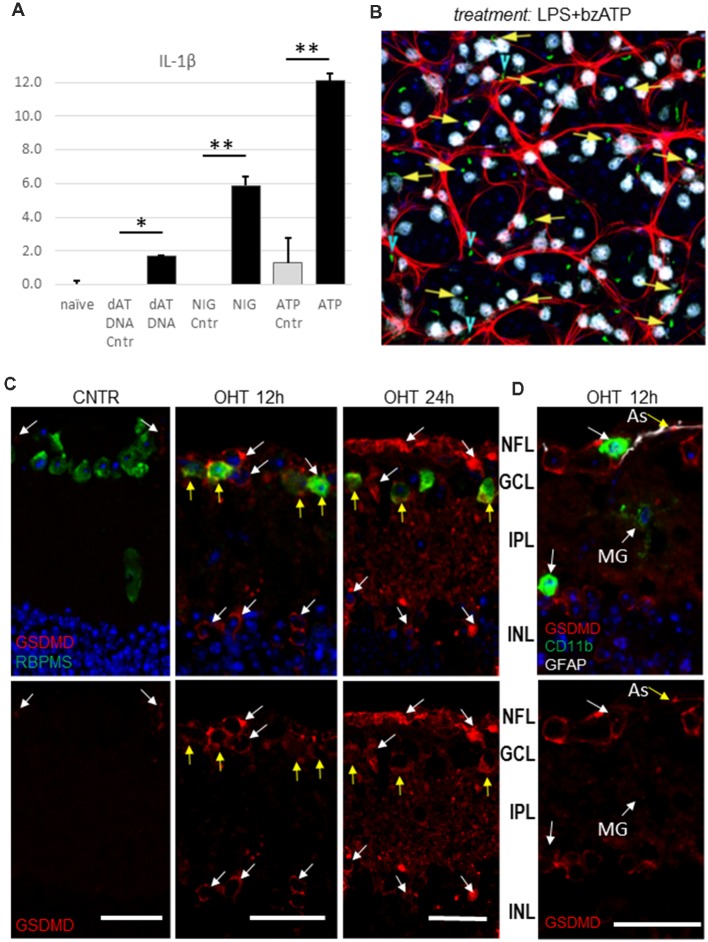
Inflammasome activation in the lipopolysaccharide (LPS)-primed retinas in response to canonical inflammasome inducers.** (A)** IL-1β release from the LPS-primed retinas challenged with canonical inflammasome inducers nigericin (NIG, 10 μM), ATP (bzATP 100 μM) and poly dA;dT DNA (dAT DNA, 15 μM) 24 h post-injection. *P < 0.05; **P < 0.02. **(B)** Representative micrograph of ASC speck (green) the ASC-citrin mouse eye, treated with LPS+bzATP mix for 24 h. Co-localization with RGCs (yellow arrows) and astrocytes (blue arrowheads) in the GCL layer of retina wholemount was analyzed using immunostaining for RBPMS (white) and GFAP (red) markers. The image is the maximal projection of a 30 μm thick confocal z-stack. **(C)** Immuno-histochemistry analysis of the pyroptotic marker protein gasdermin D expression show colocalization with RBPMS^+^ cells in the GCL (yellow arrows) at 12 h postinjury. At 24 h after OHT a considerable increase in GSDMD labeling was detected. **(D)** GSDMD labeling at 12 h postinjury showed colocalization with GFAP-positive astrocytes (As, yellow arrow), but not with microglia and macrophages (CD11b ^+^, white arrows). Bar, 25 μm on all panels.

To explore which inflammasome complexes are activated in the retina in response to distinct pro-inflammatory danger signals, we unilaterally injected cocktails of well-characterized inflammasome agonists into the vitreous body of the mouse eye without IOP elevation. The four cocktails, containing 100 ng/ml LPS mixed with 10 μg/ml nigericin (NLRP3 agonist), 2.5 mM ATP (NLRP1 and NLRP3 agonist), *S. typhimurium* flagellin 20 ng/ml (FLA-ST, an NLRC4 agonist) and Lipofectamine-conjugated poly dA:dT DNA (intracellular agonist of Aim2, 15 μM/eye), were unilaterally injected into experimental eyes with contralateral control eyes receiving vehicle only. The analysis of the ELISA data showed release of IL-1β *in vivo* in the retinas treated with these cocktails (Figure 7A). We observed the strongest release in the eyes treated with the LPS/bzATP cocktail. Importantly, this treatment triggered an early (6 h postinjection) induction of the NLRP1/NLRP3 inflammasome in RGCs and astrocytes as evidenced by the analysis of the ASC-citrin speck colocalization with RBPMS and GFAP markers, respectively (Figures 7B–D).

**Figure 8:**
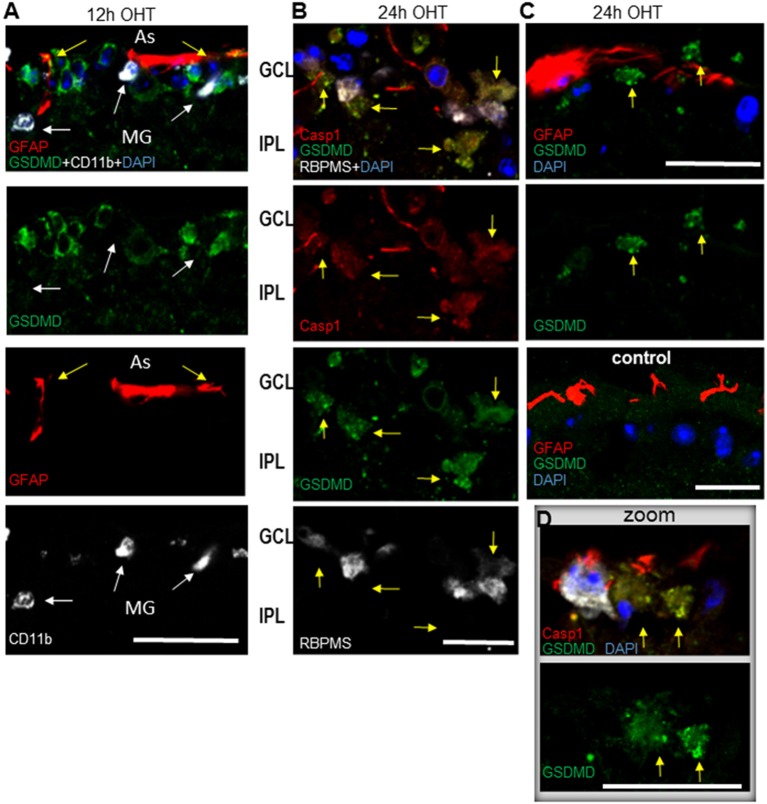
Post-OHT activation of gasdermin D (GSDMD) in the GCL.** (A)** Cytosolic labeling for gasdermin D co-localized with RGCs (RBPMS, white). At 12 h postinjury no co-localization was detected with GFAP^+^ astrocytes (As, red, yellow arrows) or CD11b^ +^ microglia (MG, white, white arrows). **(B)** At 24 h post-OHT only low-grade GSDMD^ +^ Casp1^ +^ cells with altered morphology, condensed or lacking nuclei are observed (yellow arrows), some retaining weak RBPMS marker labeling (white).GSDMD staining is mostly grainy. **(C)** GSDMD^ +^ cells (yellow arrows) are observed in close vicinity of astrocytes only in OHT injured but not in control retinas. **(D)** Inset shows a high resolution image of grainy GSDMD^ +^ labeling in neurons with altered morphology, condensed or lacking nuclei (yellow arrows) at 24 h post OHT. Astrocytes (red) in vicinity of GSDMD^ +^ neurons are highly positive for Casp1. Bar, 25 μm on all panels.

Our results, showing upregulation of three distinct canonical inflammasome complexes at 12–24 h post-OHT, raised the question whether RGC pathology in the inner retina is associated with pyroptotic cell death. The Western blot analysis of retinal extracts (Figure 1B) showed a time-dependent increase in expression and cleavage of gasdermin D (GSDMD), which is required for pyroptotic pore formation. Immunohistochemistry analysis confirmed an increase in GSDMD staining at 12–24 h postinjury (Figure 8). Importantly, RGCs were the first cell type in the GCL to become strongly GSDMD-positive at 12 h postinjury, while the labeling in astrocytes and microglia was very weak at this time point (Figure 8A). At 24 h, we detected a mild GSDMD-positive labeling of RGCs with patchy labeling of the plasma membrane that is consistent with pre-pyroptotic bubbling of the plasma membrane (Figures 8B–D). These cells were also weakly positive for Casp1 and RBPMS, and had shrunken nuclei or lacked one completely. Taken together, our results indicate that OHT injury-induced release of IL-1β occurred within hours after the insult and correlated with similarly rapid increases in gasdermin D and caspase-1 activation, first in RGCs and later in astroglia. Thus, it is reasonable to suggest that a retinal inflammasome caused Casp1-mediated pyroptotic pore formation that mediated IL-1β release have subsequently induced pyroptotic cell death in GSDMD-positive neurons.

## Discussion

In this study, we demonstrate that activation of the inflammasome and release of IL-1β in the retina occur within a few hours after OHT injury. Our evidence indicates that this acute inflammasome activation triggers pyroptotic death of RGCs *via* caspase-1-mediated mechanism.

We demonstrate that activation of the inflammasome and release of IL-1β in the retina occur within a few hours after OHT injury. We found three canonical inflammasome complexes in the retina: NLRP1, NLRP3 and Aim2, which had a distinct distribution in cell types (Figures 4, 5, Supplementary Figures S1–S3). Inflammasome activation was detected at the transcriptional level by RNAscope, at the protein level by immunohistochemistry, and at the functional level by the analysis of responses (i.e., IL-1β release) to the OHT injury or specific agonists *in vivo*. Our results are consistent with earlier studies that have detected NLRP1 and NLRP3 activation in RPE and astrocytes (Doyle et al., 2012; Tarallo et al., 2012; Chi et al., 2014; Qi et al., 2014; Albalawi et al., 2017). To the best of our knowledge, activation of NLRP1 and NLRP3 in retinal neurons are new findings. The neurotoxic activity of the Aim2 inflammasome has only been reported in the brain (Adamczak et al., 2014; Ge et al., 2018), but not in the retina. It remains to be determined, however, if activation of Aim2 which we detected in the Muller glia, plays any role in OHT injury-induced neurotoxicity. Activity of another type of inflammasome, NLRC4, was only detected by immunohistochemistry, so we did not pursue its further study.

Our immunohistochemistry data show that each type of inflammasome complex had a distinct cellular and temporal pattern of activation. In particular, the NLRP1 complex is constitutively active in RGCs, NLRP3 has low grade constitutive activity in astrocytes and vasculature but is OHT-inducible in GCL neurons, and the Aim2 complex is active in Muller glia. A certain level of constitutive activity of the three inflammasomes suggests that IL-1 signaling has a role in retinal homeostasis, as proposed in the literature (Namekata et al., 2009).

Analysis of events induced by OHT showed acute increase in inflammasome activity both in retinal neurons and glial cells in the GCL and INL layers. Which mechanism of danger signaling is the key for OHT injury-induction of inflammasomes? To this end, we show that the disruption of Panx1 signaling inhibits caspase-1 activation, suppresses and delays IL-1β release (Figures 1A, 2A). Most likely, Panx1 induces inflammasome *via* ATP release from mechanically stressed or ischemically injured cells. Purinergic signaling *via* a cell surface complex comprised from the Panx1 channel and P2X7 receptor has been implicated in inflammasome activation in the brain (Pelegrin et al., 2008; Silverman et al., 2009; de Rivero Vaccari et al., 2009) and retina (Kerur et al., 2013; Albalawi et al., 2017; Platania et al., 2017). It responds to extracellular ATP, facilitating inflammasome activation in paracrine and autocrine fashions (Reigada et al., 2008; Xia et al., 2012; Beckel et al., 2014). The fact that intravitreal injection of ATP in triggered the highest level of IL-1β release and ASC speck induction in RGCs and astrocytes (Figures 6, 7A), supports the importance of ATP-induced danger signaling for retinal inflammasome induction.

The current paradigm identifies microglia and macroglia as main drivers of neurotoxic inflammation in the retina (Hernandez, 2000; Bosco et al., 2012; Tezel et al., 2012; Abcouwer et al., 2013; Formichella et al., 2014; Mac Nair et al., 2016; Silverman et al., 2016). This model was based largely on the observed protection for RGCs against excitotoxicity and OHT-injury in mouse genetic models with suppression of glial (Ganesh and Chintala, 2011) or microglial (Silverman et al., 2016) activity or astroglial NF-kappaB signaling (Dvoriantchikova et al., 2009; Barakat et al., 2012). Contrary to this glia-centric model of neuroinflammation, our results argue that RGCs and other neurons in the GCL also directly participate in the innate immune response. Furthermore, we propose that neurons are the cell type that triggers injury response within a few hours; this occurs *via* activation of the neuronal NLRP1 and NLRP3 inflammasomes. The presence of basal NLRP1 and NLRP3 activity can prime the cells for quick response *via* the acute upregulation of Casp1 and cleavage of gasdermin D, which we detected in the inner retina after the OHT injury.

Inflammasome activation in various pathologies was shown to cause cell death *via* pyroptosis (Celkova et al., 2015; Viringipurampeer et al., 2016; Kerur et al., 2018). We have obtained evidence of caspase-1-mediated pyroptotic cell death during acute inflammasome activation and asked whether pyroptosis can contribute to neuronal death following IOP elevation? Together with the strong RGC protection by the knockout of pro-pyroptotic caspase-1, the induction of the gasdermin D pore protein in these cells supports such contribution (Figures 1C, 2C, 7B and 8). Interestingly, the strongest induction of caspase-1 and its substrate gasdermin D occurred in GCL neurons (Figures 3B, 7C, 8A). The dramatic morphological changes in RGCs spatially and temporally correlated with activation of caspase-1 and gasdermin D at 12–24 h postinjury (Figures 8B–D). These changes included nuclei shrinkage/loss in the RBPMS^+^cells with GSDMD-specific immunolabeling at the surface, which is consistent with pyroptotic death-induced membrane bubbling (Chen et al., 2016). Taken together, our results indicate that inflammasome activity-induced pyroptosis directly contributes to RGC death after transient IOP elevation.

Our discovery of pyroptosis as the early cell death type in OHT injury is highly significant because this is the most pro-inflammatory type of cell death. Pyroptotic release of ATP, ASC specks, HMGB1 and other danger signals from dying cells was shown to spread inflammation and induce secondary death of neural and blood endothelial cells (de Rivero Vaccari et al., 2014; Venegas et al., 2017; Ge et al., 2018). Retinal microenvironment changes, conditioned by pyroptotic release of these danger factors can significantly contribute the wave of secondary death both directly, *via* circulation or glial neuroinflammation. In fact, we found evidence that inflammasomal ASC specks can spread from the experimental eye into the contralateral control (normotensive) *via* blood capillaries (Figure 6A).

This study illuminates the role of neuronal inflammasome in triggering cell death. However, our data here and in earlier studies (Brambilla et al., 2009; Dvoriantchikova et al., 2009; Nikolskaya et al., 2009; Barakat et al., 2012) as well as other investigators (Tezel and Wax, 2000; Yuan and Neufeld, 2000; Ju et al., 2006; Bosco et al., 2008, 2012; Orellana et al., 2012) establish the involvement of glia and microglia in RGC pathology. It is also possible that active glia-microglia or glia-neuron signaling could facilitate neuronal pyroptosis, and indeed astrocytes and microglia are present in the vicinity of GSDMD-positive RGCs (Figures 7C, Supplementary Figure S3B). Alternatively, the timing of the observed inflammasome induction in RGCs may suggest that danger signaling, particularly ATP and ASC speck release from pyroptotic neurons can attract phagocytic microglia and astrocytes (Verderio and Matteoli, 2001; Choi et al., 2007; Brown and Neher, 2010; Chu et al., 2012; Venegas et al., 2017). An active role of such indirect signaling has been suggested in reports on hemichannel-mediated neurotoxic signaling (Bennett et al., 2012; Orellana et al., 2014) and microglial cytokine-mediated conversion of A1 astrocytes (Liddelow et al., 2017; Yun et al., 2018). These channels are particularly abundant in RGCs and were shown to facilitate OHT-induced ATP release from both glia and RGCs (Reigada et al., 2008; Xia et al., 2012; Beckel et al., 2014; Albalawi et al., 2017). Overactivation of Panx1 was shown to be essential to RGC death and retinal neuroinflammation following OHT injury (Dvoriantchikova et al., 2012, 2018). In this work, we also showed that genetic ablation or pharmacological blockade of Panx1 suppressed inflammasome activity and protected RGCs similarly to the Casp1^−/−^Casp4(11)^del^ knockout (Figure 2). We hypothesize that the initial insult kills neurons *via* pyroptosis during acute injury phase, whereas danger signaling (e.g., ATP and K^+^ release from dying neurons) facilitates activation of the retinal macroglial and microglial cells to induce secondary degeneration.

The question remains whether acute neuronal pyroptosis plays a role in the pathology of OHT injuries, including glaucoma. The IOP spike-induced degeneration of RGCs has been demonstrated in a rodent model (Joos et al., 2010). Provided that the exposure to OHT episodes was observed during the head-down posture in aging people (Ventura et al., 2013; Porciatti et al., 2017), this mechanism could potentially contribute to glaucomatous-like degeneration of RGCs.

## Author Contributions

VIS developed the central hypothesis. VIS, AP and VZS contributed to experimental design, implementation of the methodology and writing the article. AP, VIS, DP, WA, GD, ZK, JQ, GR and AR performed the experiments and the data analyses. DP and GD assisted with the research design, data interpretation, manuscript writing and editing.

## Conflict of Interest Statement

The authors declare that the research was conducted in the absence of any commercial or financial relationships that could be construed as a potential conflict of interest.
