# Supplementary material for: Inflammasome Activation Induces Pyroptosis in the Retina Exposed to Ocular Hypertension Injury
Source: Front Mol Neurosci. 2019 Mar 13;12:36. doi: 10.3389/fnmol.2019.00036 (PMC6425693; doi:10.3389/fnmol.2019.00036)
Supplement: Supplementary file 1 [file Data_Sheet_1.pdf]

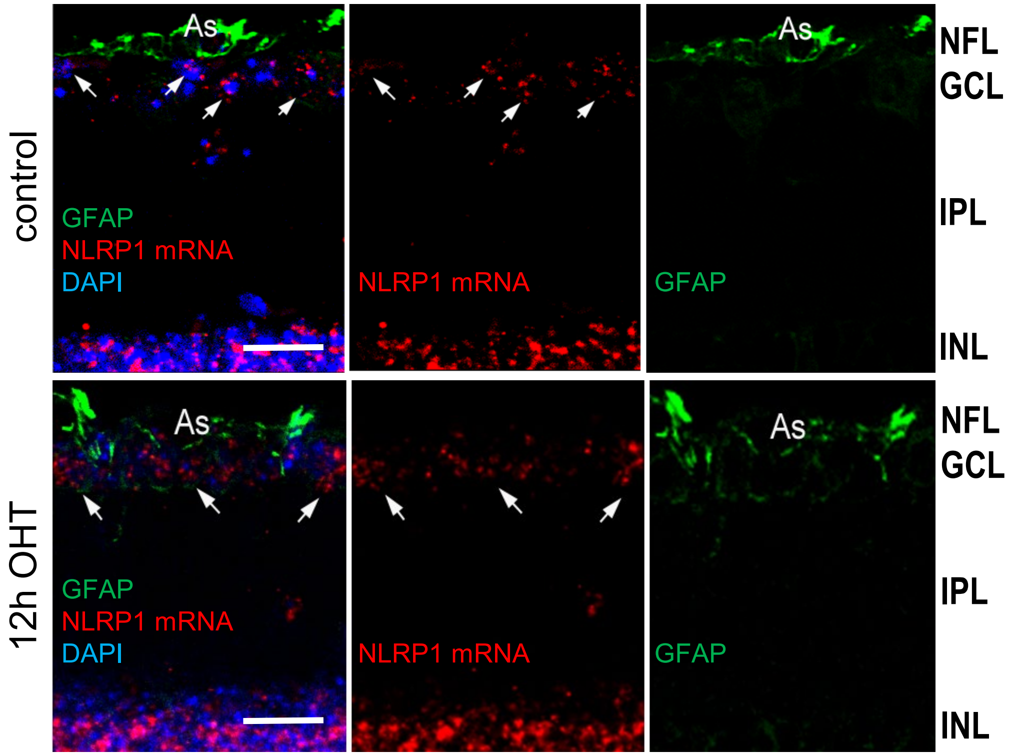

Supplement Figure 1. NLRP1 gene transcripts in the inner retina. RNAscope analysis of NLRP1 (red dots , arrows) transcript abundance in normotensive controls and experimental (OHT) retinas at 12h postinjury. Notice lack of co-localization with astrocytes (GFAP, green) in the NFL of the inner retina. Bar, 25 μm

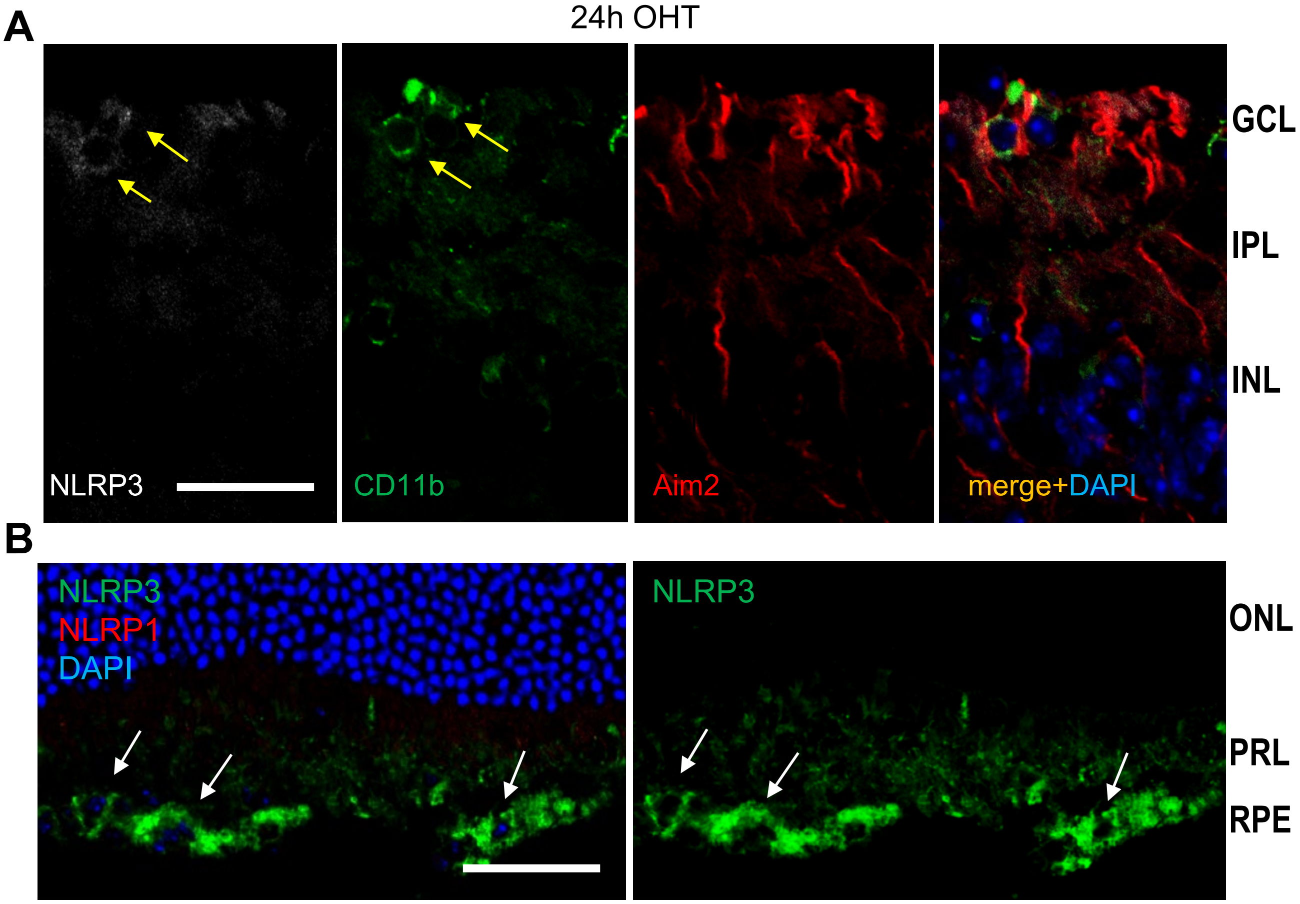

Supplement Figure 2. Cellular expression of NLRP3 and Aim2 in the retina (A) Antibody against NLRP3 (white) inflammasome protein label only CD11β^{+} microglia/macrophages (yellow arrows); Aim2 antibody (red) label only Muller glia at 12h in post-OHT. **(B).** In the outer retina, a strong NLRP3 labeling co-localizes with retinal pigment epithelial cells (arrows); no NLRP1 (red) labeling is detected.

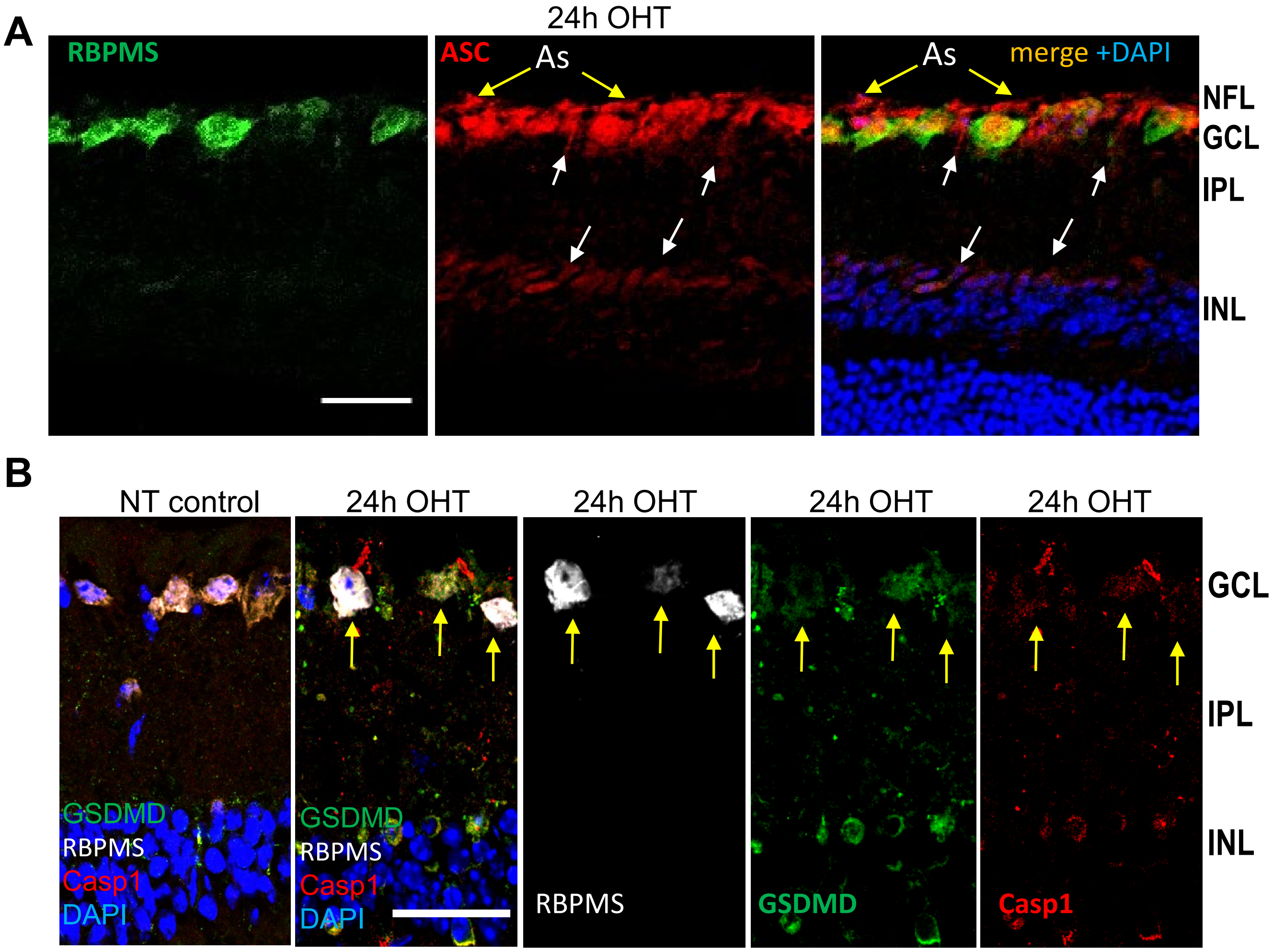

Supplement Figure 3. **Cellular expression of ASC and GSDMD proteins in the post-OHT retinas.**
Th[illegible]beling for ASC (red) show[illegible]localization with Gsdmd^{+} RGCs (green) and RBPMS-negative cells in the GCL and NFL layers at 24h post-OT injury. By cell morphology and localization, other ASC[illegible]ells are astrocytes and Muller glia (arrows). Bar, 25 µm. **(B)** GSDMD labeling in the GCL co-localizes (yellow arrows) with Casp1 staining predominantly in RGCs (RBPMS) at 24h post-OHT. In the INL, GSDMD labels a subpopulation of RBPSM-negative neurons also showed. Bar, 25 µm on all panels

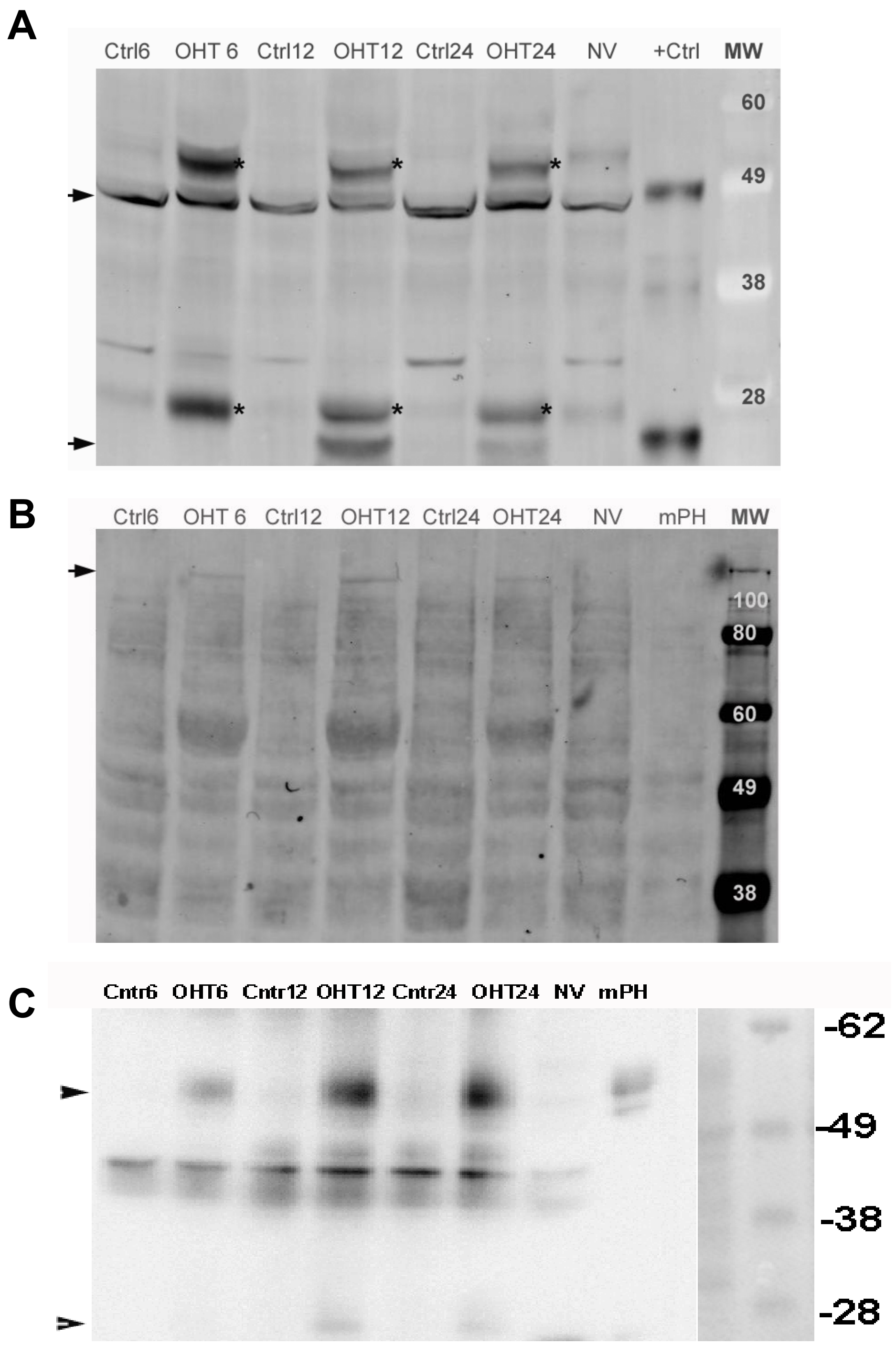

Supplement Figure 4. Untrimmed Western blot images for Casp1 NLRP3and.

A. Western blot of vitreo-retinal lysates from OHT-challenged (OHT), normotensive control (Cntr) and naïve (NV) eyes probed with Caspase-1 antibodies (p20 Casper-1). Extracts from LPS-activated bone marrow mouse macrophages, treated with nigiricin served as positive controls (+Ctrl).
B. Same blot probed with antibodies against NLRP3 sensor protein.
C. Western blot of vitreo-retinal lysates from OHT-challenged (OHT) eyes , probed with antibodies against GSDMD protein
